# Species-specific wiring of cortical circuits for small-world networks in the primary visual cortex

**DOI:** 10.1371/journal.pcbi.1011343

**Published:** 2023-08-04

**Authors:** Seungdae Baek, Youngjin Park, Se-Bum Paik

**Affiliations:** 1 Department of Bio and Brain Engineering, Korea Advanced Institute of Science and Technology, Daejeon, Republic of Korea; 2 Department of Brain and Cognitive Sciences, Korea Advanced Institute of Science and Technology, Daejeon, Republic of Korea; University of Osnabrück: Universitat Osnabruck, GERMANY

## Abstract

Long-range horizontal connections (LRCs) are conspicuous anatomical structures in the primary visual cortex (V1) of mammals, yet their detailed functions in relation to visual processing are not fully understood. Here, we show that LRCs are key components to organize a “small-world network” optimized for each size of the visual cortex, enabling the cost-efficient integration of visual information. Using computational simulations of a biologically inspired model neural network, we found that sparse LRCs added to networks, combined with dense local connections, compose a small-world network and significantly enhance image classification performance. We confirmed that the performance of the network appeared to be strongly correlated with the small-world coefficient of the model network under various conditions. Our theoretical model demonstrates that the amount of LRCs to build a small-world network depends on each size of cortex and that LRCs are beneficial only when the size of the network exceeds a certain threshold. Our model simulation of various sizes of cortices validates this prediction and provides an explanation of the species-specific existence of LRCs in animal data. Our results provide insight into a biological strategy of the brain to balance functional performance and resource cost.

## Author summary

The brain performs visual object recognition using much shallower hierarchical stages than artificial deep neural networks employ. However, the mechanism underlying this cost-efficient function is elusive. Here, we show that cortical long-range connections (LRC) may enable this parsimonious organization of circuits for balancing cost and performance. Using model network simulations based on data in tree shrews, we found that sparse LRCs, when added to local connections, organize a small-world network that dramatically enhances object recognition of shallow feedforward networks. We found that optimization of the ratio between LRCs and local connections maximizes the small-world coefficient and task performance of the network, by minimizing the total length of wiring needed for integration of the global information. We also found that the effect of LRCs varies by network size, which explains the existence of species-specific LRCs in mammalian visual cortex of various sizes. Our results demonstrate a biological strategy to achieve cost-efficient brain circuits.

## Introduction

**Fig 1:**
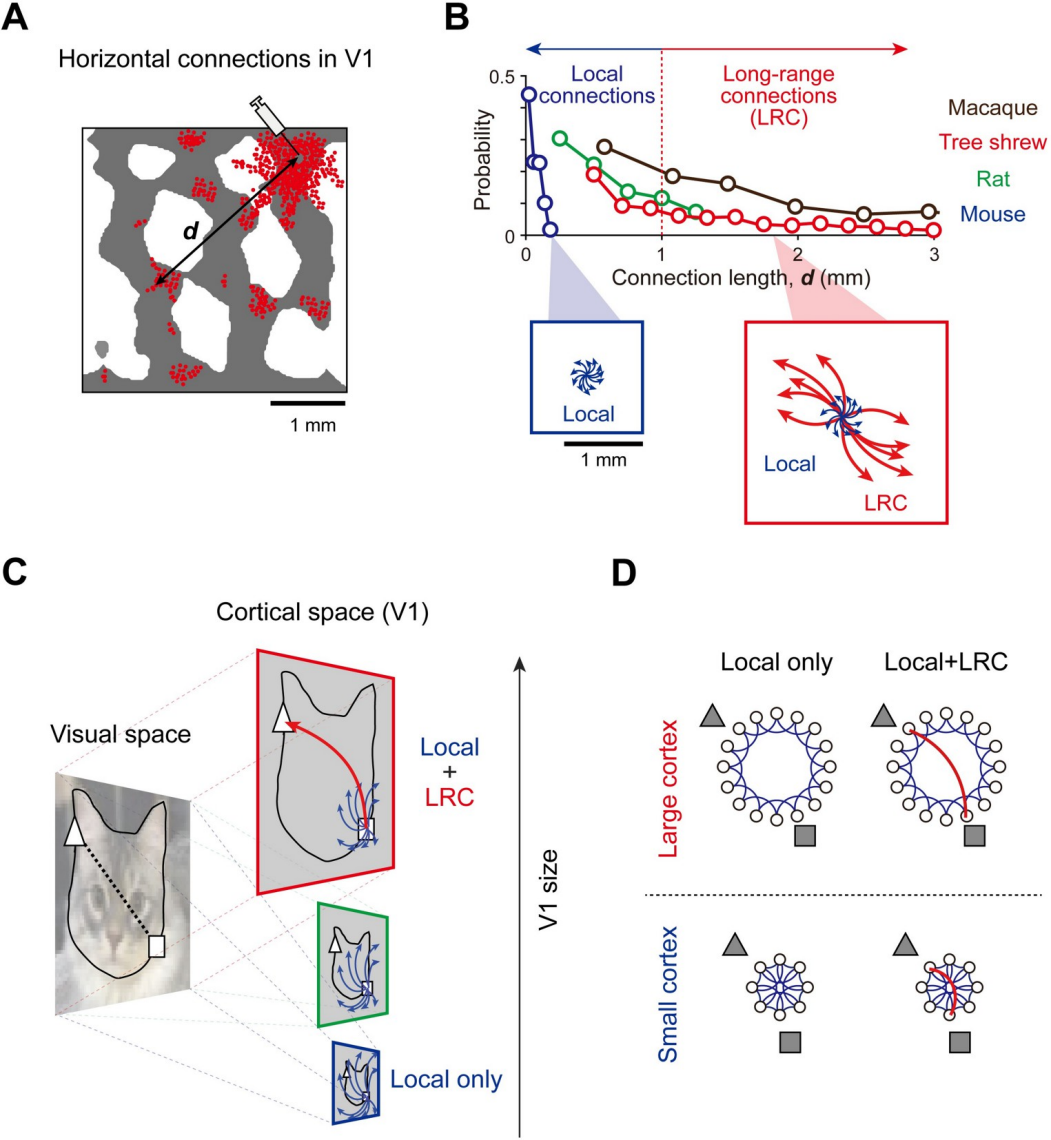
Species-specific existence of long-range horizontal connectivity in V1. (A). Illustration of long-range horizontal connections observed in the primary visual cortex (V1) of tree shrews (following Bosking 1997). Red dots represent synaptic boutons of lateral connections with retrograde labeling by injection. (B). Illustration of distribution of the connection lengths of lateral connections in various species (following Seeman 2018 and Bosking 1997). LRCs are not found in species with a small V1 area, such as mice. (C). Illustrations of retino-cortical projections in species with different magnification factors. According to the size of the cortex, the same object in the visual space can cover different sizes of the spatial area in the cortical space. (D). LRCs may act as shortcuts for inter-neural communications in a large V1 network but not in a small network, where local connections suffice.

Long-range horizontal connections (LRCs, Fig 1A) are characteristic anatomical structures observed in the primary visual cortex (V1) of various mammalian species, such as monkeys [1,2], cats [3,4], tree shrews [5,6], gray squirrels [7], ferrets [8], and rats [9,10]. Given their extraordinarily long wiring (up to 2–3 mm), LRCs are distinguished from local connections of a short lateral spread (up to 1 mm) [11–15]. Given the argument that the brain has evolved to develop its structure by balancing wiring costs and functional efficiency [16–20], it may be disadvantageous to develop such connections with high structural costs [21–23] unless they play a crucial role in information processing. Previous studies have suggested possible roles of LRCs that offset their high wiring cost, such as the amplification of the weak feedforward input, the enhancement of object contour detection, or early-stage contextual modulation [24–26], but the exact functions of LRCs for visual information processing are still elusive.

Notably, LRCs are not found in species with a small V1 area, such as mice (Fig 1B). Mice and rats are genetically close relatives with neuroanatomical similarities of the functional circuits in V1 [27–29]. However, rats [9] have LRCs clearly distinct from their local lateral spreads, whereas mice [30] do not. Both on an absolute scale (Fig 1B) and on a scale normalized to the size of V1 in each species (S1 Fig), LRCs longer than 10% of the V1 length are consistently observed in many species, while they do not exist in mice. This discrepancy among different species may provide hints about the conditions under which the development of LRCs is advantageous for information processing, offsetting their high wiring cost.

An important clue may be the differences in magnification factors between species with and without LRCs. For an input that covers the same size of the visual space, the size of the cortical space that matches this input is fairly different across species (Fig 1C). This indicates, for example, that the same distance in the visual space would be mapped approximately ten times further in the cortical space of tree shrews compared to that of mice (magnification factor; mice: ~50 deg/mm, rats: ~20 deg/mm, tree shrews: 4~4.5 deg/mm). Then, in a large V1 network (Fig 1D, large cortex), short local connections would not suffice to integrate distant visual features and LRCs can act as shortcuts for cortical inter-neural communications. This is in contrast to the condition of a small V1 (Fig 1D, small cortex), where local connections are sufficient to integrate any arbitrary location in V1. Considering that the visual information of natural images contains a wide range of spatial frequency components [31], the integration of long-distance or low-spatial-frequency features may be one of the important roles of LRCs, particularly in a large-scale network.

In this scenario, important questions arise—Does this model account for the species-specific existence of LRCs? What is the threshold of the network size where LRCs become advantageous while also compensating for their high wiring cost? What determines the proper ratio between LRCs and local connections? To answer these intriguing questions, here we introduce the idea that the biological structure of the V1 circuit can be described as a “small-world network” [32–34] in terms of its wiring profile. A “small-world” can be defined as a network that minimizes the average “global” distance between distant nodes while maximizing “local” interactions between adjacent nodes, with a limited number of links. According to the theory, there exists an optimal condition in which two contradictory goals, the minimum wiring cost and the maximum performance, can offset each other. Mathematically, this can be achieved by dense local clusters with sparse, long shortcuts for a sufficiently large network or can be achieved solely by localized wirings when the network is small enough. From this analogy, we hypothesized that local connections and LRCs develop to organize a “small-world network” optimized to each network size. We assumed that the small-world coefficient of each network predicts the ability to encode visual information on a wide spatial frequency spectrum (from the local to global scale) to find the optimal wiring structure of neural circuits depending on the network size.

To validate this theoretical idea, we implemented a model network of the retino-cortical pathway and simulated various circuit wiring conditions while the network performs image classification tasks. First, in a large network, we found that the clustering coefficient (C) of the network is strongly correlated with its ability to encode “local” information of visual stimuli, while the average path length (L) is correlated with the network’s ability to recognize distant “global” features. Overall, the small-world coefficient, maximized by a high C and low L, appeared to be tightly correlated with the performance of the network on the classification of images containing both local and global information as natural images.

Further, we tested whether the organization of LRCs and local connections optimized to become a small world can also be achieved from a random network by balancing the network performance and the wiring cost. We trained a randomly initialized network with a cost function that contains both performance and wiring cost terms. We found that a significant amount of LRCs survived after training in spite of the wiring cost penalty such that the ratio between the number of LRCs and the local connections converges to a constant value.

Notably, for a small network, we could not observe such effects of LRCs—adding LRCs to local connections does not increase the classification performance of networks because the small-world coefficient of the network circuit is not affected, as predicted by our model. This result demonstrates the network-size-dependent contribution of LRCs to the small-world coefficient organization and explains the species-specific existence of LRCs in mammalian brains according to the size of the cortex in each species. Taken together, our results provide a theoretical framework by which to understand the emergence and development of optimal architectures of the cortical circuitry in the brain.

## Results

### Long-range connections for integration of distant visual information

**Fig 2:**
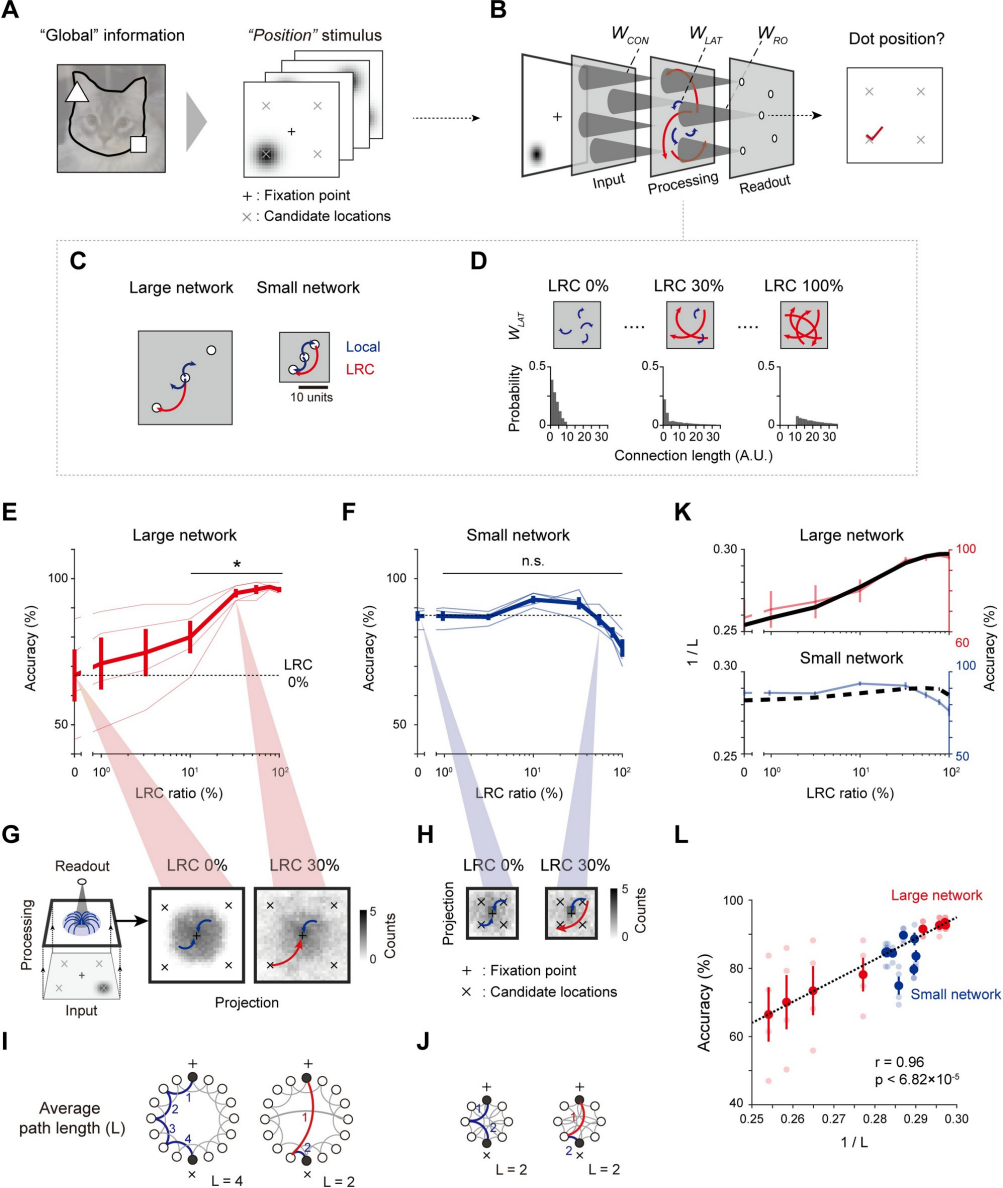
Integration of global information by LRCs in a large network. (A). Design of a visual stimulus containing “global” information in the form of positions of dots. (B). A simplified three-layer network model of the early visual pathway. (C). Two distinct sizes of the model network used in the simulations. (D). LRC ratio, i.e., the number of LRCs relative to the total number of connections, varying from 0% to 100%, while the total number of connections remains constant. The connection length in the simulation was sampled from observed statistics in biological data [5]. (E)-(F). The classification accuracy for the “position” dataset under variations of the LRC ratio in large and small networks. Thin lines indicate the results for different conditions of the center-dot distance in the stimulus (See **S2A Fig** for details). Bold lines indicate performance averaged over all stimulus conditions. (G)-(H). Visualization of the connectivity projection from the processing layer to a single readout unit in a large and a small network. The gray level of each pixel represents the number of connections linked to a readout neuron, and the total grayscale area indicates the effective “recognition” range of the readout. (I)-(J). Illustration of the average path length L of the network. Note that a wide effective range of lateral connections, leading to a small L, is necessary to identify global positional information in the visual stimulus. (K). Similarity between the modulation of the value of 1/L and of the classification accuracy of the network during the rewiring process when varying the LRC ratio. (L). A strong correlation is observed between 1/L and the classification accuracy of the network regardless of the network size and/or the stimulus condition. For each value of 1/L on the x axis, transparent dots indicate the results for different conditions of the center-dot distance in the stimulus and bold dots indicate the result averaged over all stimulus conditions. Error bars represent the confidence interval for 20 trials.

To investigate the contribution of long-range connections (LRCs) for the integration of visual information in V1, we designed a three-layer convergent neural network as a simplified model of the early retino-cortical visual pathway (Fig 2A and 2B). The model network consists of an input layer (retina), a hidden processing layer (V1), and a readout layer (higher visual areas) that perform a simple image classification task [35]. Model neurons in the layers are inter-connected via three different types of feedforward (inter-layer) projections and lateral (intra-layer) wirings—(i) The inter-layer feedforward connections from the input to the hidden layer (*W*_*CON*_) consist of local convergent projections, following previous observations in the early visual pathway [36]. (ii) Feedforward projections from the hidden to the readout layer (*W*_*RO*_) also have a local convergent structure, following the observation that retinotopy is maintained up to higher visual areas [37] in the brain. Similar to the viewpoint fixation commonly applied in behavioral experiments [38,39], readout neurons are selected such that their receptive field is located at the fixation center of the visual space and that projections to the readout layer cover the entire input visual field. (iii) The intra-layer lateral connections (*W*_*LAT*_), the main control parameter of the simulation, are modeled to have the length sampled from the observed distribution of wiring in tree shrews [5] (S1 Fig, See Materials and Methods for details).

First, to investigate whether LRCs enhance the integration of global information, i.e., long-distance correlations contained in visual images, we designed a stimulus dataset (32 × 32 pixels) in which only the position of an object varies (Fig 2A). Specifically, the “position” dataset consists of images with a dot located in one of the quadrants and a label that match to each location (NE, NW, SE, SW). Then, the network was trained with 10,000 images to classify the dataset according to the dot positions, and 2,000 novel images were used as the test dataset (Fig 2B). Using two distinct sizes, which indicate the area of the hidden layer, of the model network (Fig 2C, large network: 32 × 32 units, small network: 17 × 17 units), we examined the change in the classification performance while varying the ratio of LRCs in the constant total number of lateral connections (Fig 2D). In the large network, we found that the classification performance increases as the ratio of the LRCs increases (Fig 2E, n = 20 randomly initialized networks, one-side rank sum test, *p < 1.35×10^−3^). On the other hand, in the small network, the change in the performance with the addition of LRCs was not significant (Fig 2F, n = 20, one-side rank sum test, n.s., p = 0.56). This occurred because an object in the visual space is mapped as different sizes in the cortical space depending on the size of the network. In a small network, local connections are sufficient to integrate distant visual features due to the relatively small cortical magnification factor. On the other hand, the same object may not be integrated only with local connections in a large network unless LRCs are added and thus also contribute. To visualize this, we examined the connectivity projection from the processing layer to the readout neurons by visualizing the spatial distribution of the connection weights between the processing layer and each readout neuron (Fig 2G and 2H). We found that pure local connections were insufficient to enable the readout at the center to integrate the information of object locations at the periphery visual space (Fig 2G, LRC 0%). However, the addition of a small portion (~30%) of LRCs could dramatically expand the range of the spatial reach-out of the readout neuron so that the location of the dot stimuli could be successfully recognized (Fig 2G, LRC 30%). In contrast, the addition of LRCs in a small network did not induce such a dramatic change, as pure local connections already suffice to encode the entire visual space (Fig 2H). We confirmed that this tendency was consistent under variations of the center-dot distance (S2A and S2B Fig).

This result suggests that LRCs can contribute to the encoding of the long-range features contained in visual images when pure local connections cannot fully integrate the long-distance spatial correlations of the visual components, particularly with a large cortical layer. In this case, LRCs may serve as a shortcut in the circuit (Fig 2I and 2J), and this type of circuit modulation can be quantified by the average path length (L) [34] between two random neurons in the layer. Specifically, we hypothesized that the addition of LRCs can reduce the average path length L and that the reduced L may enhance the ability of the network to encode long-range positional information. To validate this hypothesis, we measured the average path length of the network while varying the LRCs ratio and then compared it to the classification performance observed in each network condition (Fig 2K). We found a strong correlation between the network performance and the reciprocal of the path length (1/L) regardless of the conditions of the stimulus images (center-dot distance variations) or of the networks (layer size variations) (Fig 2L, n = 20, Pearson correlation coefficient, Overall, r = 0.96, p < 6.82×10^−5^; Large network, r = 0.99, p < 3.64×10^−6^; Small network, r = 0.58, p < 7.73×10^−3^). This result suggests that the average path length can be an indicator of the network’s capability to integrate such long-distance spatial correlations or “global” information contained in the visual stimulus.

### Network circuits for encoding various spectrums of a visual stimulus

**Fig 3:**
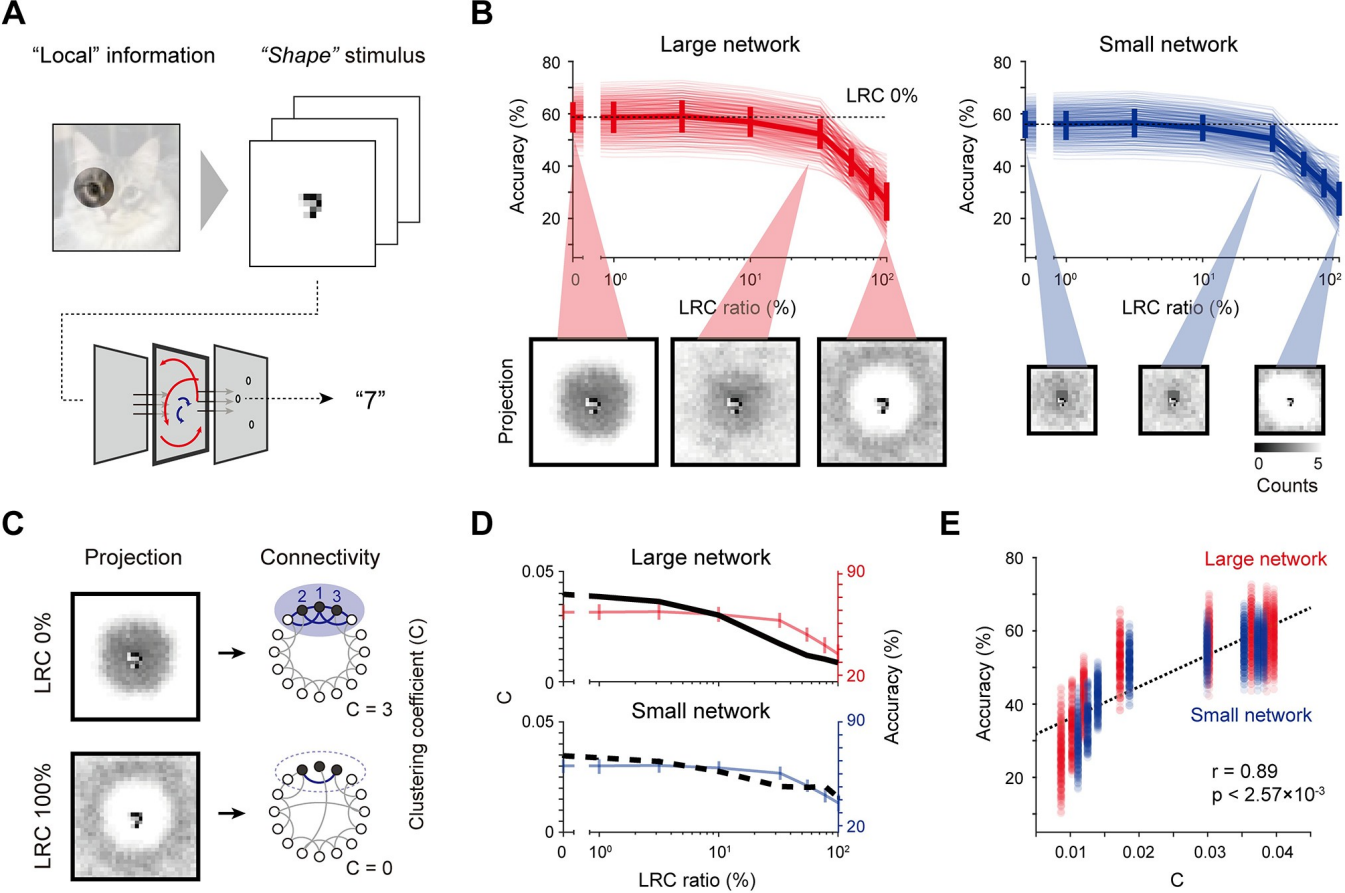
Local connections are required to integrate local information in a large network. (A). Design of a visual stimulus containing “local” or “shape” information with an MNIST digit located in the center. (B). Classification accuracy for the “shape” dataset with variations of the LRC ratio and of the network size. For both large and small networks, the performance decreased as the LRC ratio was increased. Thin lines indicate the individual network performance outcomes under variations of the stimulus parameters, and bold lines indicate the averaged performance (See **S2C Fig** for details). (C). Illustration of the clustering coefficient C of the network. Note that a high connection density, leading to high C, is necessary to recognize the detailed shapes of the digits. (D). Similarity between the modulation of the value of C and of the classification accuracy of the network during the rewiring process when varying the LRC ratio. (E). A strong correlation is observed between C and the classification accuracy of the network regardless of the network size and/or the stimulus condition. For each value of C on the x axis, transparent dots indicate the results for different combinations of digits in the stimulus and bold dots indicate the result averaged over all stimulus conditions. Error bars represent the confidence interval for 20 trials.

In natural circumstances, visual inputs normally contain a wide range of frequency components [31]. To examine the relationship between the organization of network circuits and the encoding of visual frequency spectrums, we prepared a new dataset by modifying MNIST images (Fig 3A), each of which contains distinct components of the spatial correlation (“shape,” “position” and “both”)—Classification of “shape” stimuli demand the encoding of local profiles such as the shape of the digits, while the “position” stimuli require distinguishing global features such as the positional relationship between two distant stimuli. “Both” stimuli contain both local and global information so that classification in these cases requires the encoding of a wide visual spectrum similar to that of natural images. Using these datasets, we trained the networks and examined the classification performance while modulating the network circuits by varying the ratio of the LRCs.

In a classification task using the “shape” dataset, we found that the performance decreased as the LRC ratio increased (Fig 3B, top and S2C and S2D Fig) for both large and small network conditions. When the network consists of 100% local connections, the receptive field (i.e., spatial distribution of the processing layer units connected to a readout unit) of each readout neuron overlapped considerably with the center area of the shape stimulus (Fig 3B, bottom), leading to the successful classification of the stimulus digits. On the other hand, as the ratio of LRCs in the network increased, the connection density in the local center area was reduced while the range of the projection to a readout neuron widened to the periphery area, leading to a reduction in the classification performance. With this observation, we assumed that the clustering coefficient (C) [34], which represents the degree of local clusters in the network connectivity, can predict performance for the shape stimulus (Fig 3C). We measured the clustering coefficient of each network while varying the LRC ratio (Fig 3D) and compared these outcomes to the classification performance of the “shape” dataset. As expected, a strong correlation between the clustering coefficient C and the performance for the “shape” dataset was found (Fig 3E, n = 20, Pearson correlation coefficient, Overall, r = 0.89, p < 2.57×10^−3^; Large network, r = 0.90, p < 5.32×10^−3^; Small network, r = 0.89, p < 4.57×10^−3^) regardless of the network size.

**Fig 4:**
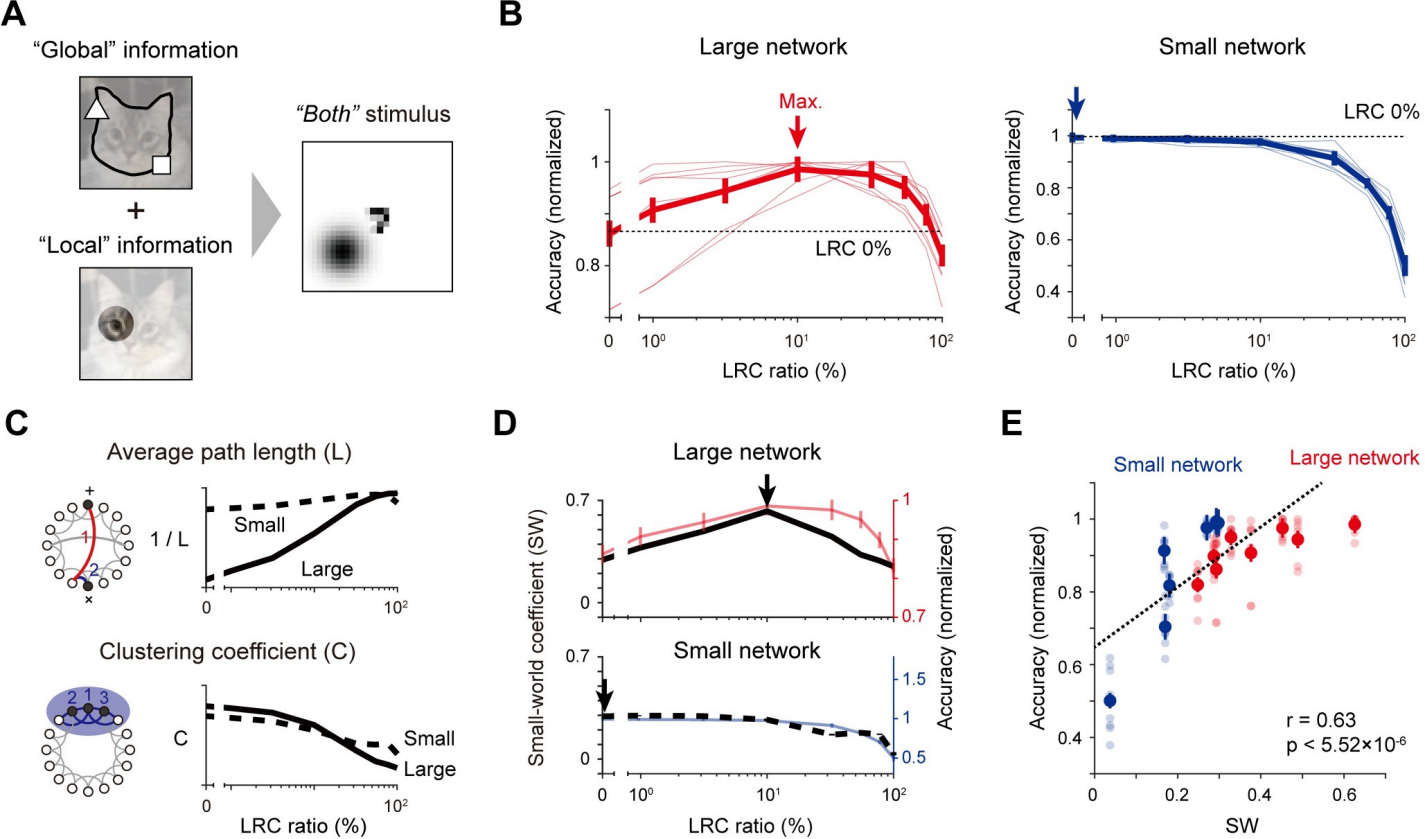
LRCs organize a small-world network to enable the recognition of various visual features. (A). Design of visual stimulus containing both “global” and “local” information with an MNIST digit in the center and a dot located in one of the quadrants. (B). Classification accuracy with variations of the LRC ratio. The large network (left) showed the best performance with a combination of sparse (10%) LRCs and dense local connections, while the small network (right) achieved its best result with local connections only. Arrows indicate the condition of the best performance. Thin lines indicate the individual network performance under variations of the stimulus parameters, and bold lines indicated the averaged performance (See **S3 Fig** for details). (C). Similarity and differences in the modulation of L and C during LRC ratio variations in large and small networks. Note that L changes significantly due to the modulation of the LRC ratio in a large network, but not in a small network, while C changes similarly in both small and large networks. (D). Similarity between the modulation of the small-world coefficient (SW) and the classification accuracy of the network during the rewiring process when varying the LRC ratio. (E). A strong correlation is observed between SW and the classification accuracy of the network regardless of the network size and/or the stimulus condition. For each value of SW on the x axis, transparent dots indicate the results of different combination of digits and dot positions in the stimulus, and bold dots indicate the result averaged over all stimulus conditions. Error bars represent the confidence interval for 20 trials.

Lastly, we investigated the circuit-structure-dependent performance of the network using the “both” dataset that contains local and global feature components simultaneously, similar to natural images (Fig 4A). Distinct from the two previous conditions, the performance curve showed significantly different characteristics in the two sizes of networks. The large network performed best with a combination of sparse (10%) LRCs and dense local connections, while the small network achieved maximum performance accuracy with only a circuit of local connections (Fig 4B). These trends were consistent with variations of the stimulus parameters (S3 Fig). Notably, these changes in the performance curve upon circuit structure variations corresponded with those in the small-world network architecture [32–34] of the network circuit. This is a theoretically understandable result considering that a small-world network is composed of high local clustering and a short average global path length [34]. This condition can be achieved from dense local clusters along with sparse long shortcuts when the network is large enough, whereas the equivalent condition can be solely achieved by localized connections only in a small network (Fig 4C), particularly because the average path length does not significantly change with the addition of long-range shortcuts in a small network. We measured a correlation between the small-world coefficient (SW) and the classification performance of network at each size (Fig 4D). Across various conditions of network parameters (e.g., the layer size and length distribution of lateral connections) and stimulus features (e.g., the choice of center digit and the distance between the center digit and the dots), we found that the small-world coefficient shows a significant correlation with the performance of the network for the “both” dataset (Fig 4E, n = 20, Pearson correlation coefficient, Overall, r = 0.63, p < 5.52×10^−6^; Large network, Large network, r = 0.53, p < 4.43×10^−5^; Small network, r = 0.89, p < 5.73×10^−8^). This suggests that the combination of sparse LRCs and dense local lateral connections composes a small-world network that enables consistent visual encoding for stimuli of a wide visual frequency spectrum matching that of natural images.

### Small-world coefficient of the network predicts the size-dependent effect of LRCs for visual encoding

**Fig 5:**
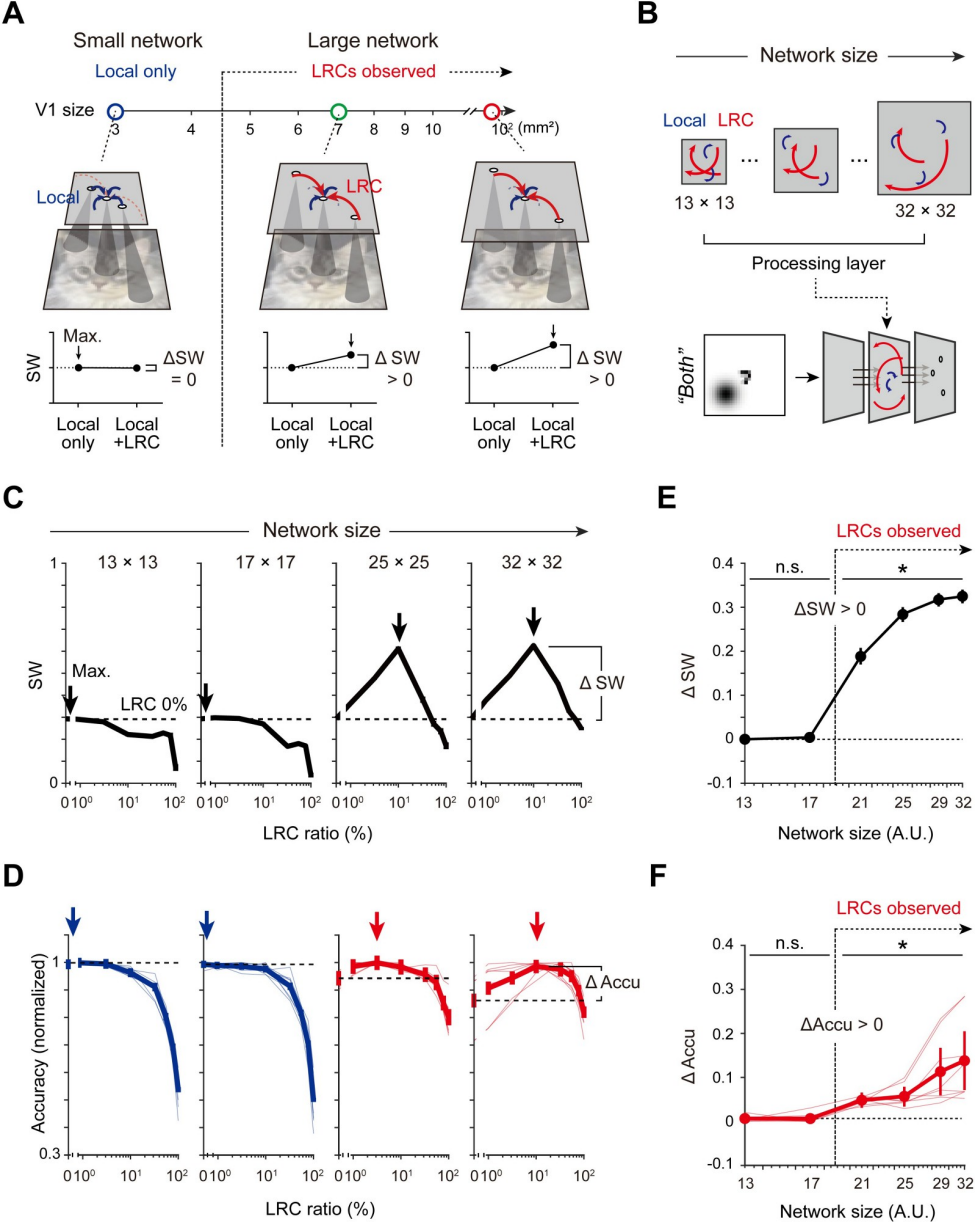
Small-world coefficient of the network predicts the size-dependent effect of LRCs for visual encoding. (A). A hypothetical scenario for the network-size-dependent existence of LRCs. The species-specific emergence of LRCs is explained by the size-dependent enhancement of small-world coefficient (ΔSW) with LRCs. (B). To validate the hypothesis, the small-world coefficient (SW) and the performance of the network were examined while the network size and the LRC ratio varied. (C). Change in the SW of the network as a function of the LRC ratio with different sizes of networks. Arrows indicate the maximum value point and dashed lines indicate the value of SW with 0% LRCs. ΔSW is defined as the difference between the two values, as illustrated. (D). Modulation of the normalized classification accuracy (ΔAccu) as a function of the LRC ratio with different sizes of networks. ΔAccu is defined as the difference between the maximum performance and that with 0% LRCs. (E)-(F). Changes of ΔSW and ΔAccu as a function of the network size. Note that both ΔSW and ΔAccu become positive only when the network size exceeds a certain threshold. Thin lines indicate the performance under the variation of local and global parameters of the stimulus (See **S3 Fig** for details). Bold lines indicated the averaged performance during variation of the stimulus. Error bars represent the confidence interval for 20 trials.

We undertook a further investigation of the layer-size-dependent contribution of LRCs to the small-world coefficient of a network and whether this can explain the existence of LRCs across different species (Fig 5A). Under the assumption that the process of early visual encoding in the retino-cortical pathway, particularly for the integration of the spatial correlation of stimulus information, can be described by our simplified three-layer model, we hypothesized that the existence of LRCs may depend on whether the small-world coefficient of the neural circuit can be enhanced by LRCs in each cortical condition; if the size of the network is small, as in mice, introducing LRCs would not change the small-world coefficient or stimulus classification performance (Fig 5A, ΔSW = 0). In contrast, when the size of network becomes larger, the addition of LRCs introduces inter-neuronal shortcuts and enables communication by distant neurons, eventually increasing the small-world coefficient and performance of the network (Fig 5A, ΔSW>0).

To validate this hypothesis, we performed a connectivity analysis of networks of various sizes, along with an image-classification test of the “both” dataset (Fig 5B). We measured changes in the small-world coefficient (ΔSW) and performance (ΔAccu) of the network as the difference between the initial and the maximum value and investigated the corresponding relationship between these values and the network size. When the model network size was large, as predicted, the introduction of LRCs increased both the classification accuracy and the small-world coefficient (Fig 5C and 5D, 32 × 32). In contrast, when the network size was smaller than 17 × 17, the performance and small-world coefficient did not increase further regardless of the amount of LRCs added (Fig 5C and 5D, 13 × 13). Importantly, the enhancements of the performance (ΔAccu) and small-world coefficient (ΔSW) by LRCs were positive only when the network size exceeded a certain threshold (Fig 5E, ΔSW > 0, n = 20, one-sided rank-sum test, p < 0.05; Fig 5F, ΔAccu > 0, n = 20, one-sided rank-sum test, p < 0.05). This result demonstrates the network-size-dependent contribution of LRCs to the modulation of small-world coefficient, which provides a possible explanation for the species-specific existence of LRCs in mammalian brains of various sizes.

### Conditional development of LRCs for balancing the performance and the wiring cost

**Fig 6:**
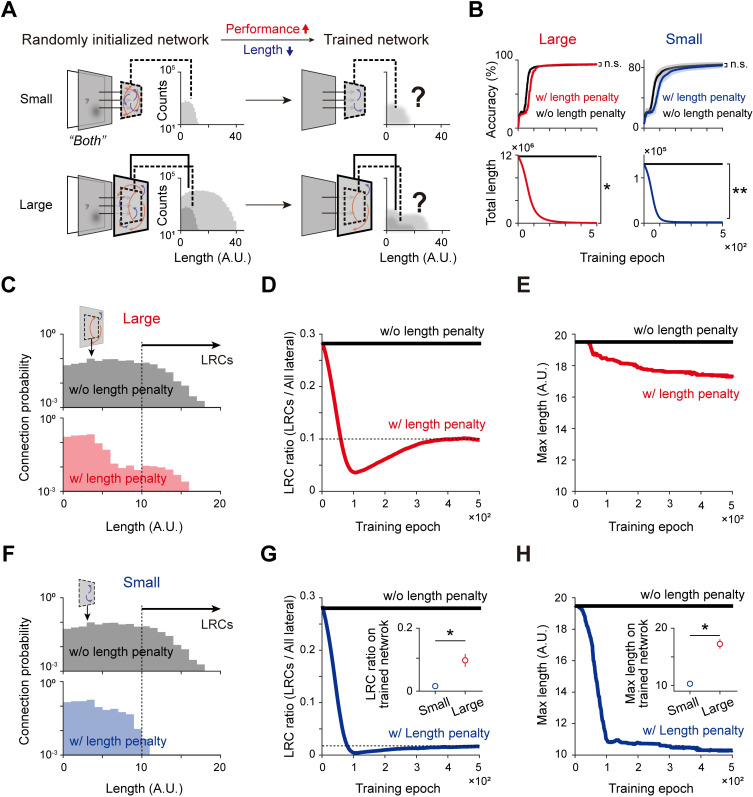
Emergence of LRCs for size-dependent optimization of the performance and wiring cost. (A). Networks of two different sizes were trained and pruned from random initial wiring conditions to find the optimal condition that maximized the classification performance at the minimal wiring cost. (B). The classification accuracy increased and the total wiring length decreased in both networks, as intended by the new loss function that consists of classification error and length-penalty terms. (C). Distribution of the lateral connection length in a large network after training, with (red) and without (black) a length penalty. (D). A certain portion of LRCs survived after training even with a length penalty. Note that the LRC ratio quickly decreases initially but then converges asymptotically to a constant value. (E). The maximum length of surviving connections also converges to a certain value above the LRC threshold (10 units). (F). Distribution of the lateral connection length in a small network after training, with (blue) and without (black) a length penalty. (G)-(H). LRCs scarcely survive after training in a small network. The LRC ratio and the maximum length of connections after training are significantly lower than those of a large network (insets). Error bars represent the standard deviation for 20 trials.

Thus far, we have shown that LRCs added to a large network can enhance the network’s ability to integrate visual information by modifying the circuit structure to organize a small-world network. At this point, we re-confirm our findings from the opposite direction: Can LRCs develop spontaneously from random initial wirings by striking a balance between the performance and the wiring cost at each size of the network? (Fig 6A). To answer this question, we designed a new loss function [35], $Loss=(1-B)\times E_{error}+B\times E_{length}$, which consists of two independent components–the classification error (*E*_*error*_) and the length-penalty (*E*_*length*_) terms. The classification error term is supposed to force the network to improve its classification performance accuracy, while the length-penalty term is to minimize the total wiring length in the network. Using this new loss function and with the “both” stimulus dataset, we trained randomly initialized networks, with connections weakened below a set threshold (|w|<0.05) pruned at each training epoch. Lastly, retained connections in the small and large networks were compared. For a fair comparison, connections in the center area, identical in size to the small network, were sampled from the large network (Fig 6A, dashed squares).

While varying the relative weight between the two loss terms (S4 Fig), we found a fairly large parameter regime in which the network can be trained to increase the classification performance while the total connection length decreases simultaneously. We observed that even with the additional term of the length penalty, there was no significant degradation of the final classification accuracy in both the large and small networks (Fig 6B, Large networks, n = 20, two-sided rank-sum test, n.s., p = 0.46, *p < 6.81×10^−8^; Small networks, n = 20, two-sided rank-sum test, n.s., p = 0.12, *p < 6.80×10^−8^). Interestingly, a certain portion of very long connections still survived at the end of the training despite the strong pressure by the length penalty (Fig 6C). We observed that the ratio of LRCs dropped sharply at the early stage of the training but asymptotically approached a constant, nonzero value instead of converging to zero (Fig 6D). The maximum length among all surviving connections also converged to a certain value above the LRC threshold (Fig 6E), reduced by only 10% compared to that in the absence of the length penalty. In additional simulations using the “shape” and “position” datasets (S5 Fig), we found that a proportion of the LRCs survived only when the input data contained “global” information (only for the “position” and “both” datasets and not for the “shape” dataset). These results demonstrate that LRCs in large networks may be the result of a structural optimization for the cost-efficient handling of inputs with global information.

However, such LRCs did not emerge in a small network. We found that the connectivity distribution after training for the optimization of the performance appeared differently in the small and large networks. While the length distribution of the large network model showed a significant portion of surviving LRCs (Fig 6C), the small network model contained almost no LRCs (Fig 6F and 6G). After the training, the ratio of LRCs in the small network was significantly lower than that in the large networks (Fig 6G, inset, n = 20, two-sided rank-sum test, *p < 6.80×10^−8^). We also confirmed that the maximum connection length in the small network dropped during training to a value significantly lower than that in large networks (Fig 6H, inset, n = 20, two-sided rank-sum test, *p < 5.39×10^−8^). Additionally, when the network sizes varied continuously from 17x17 to 32x32, we found that the ratio of LRCs and the maximum connection length increased as the network size increased (S6 Fig). This tendency was observed consistently during variations of the network structural parameters (S6 Fig). Then, in a closer examination of whether the emergence of LRCs is determined by the number of neurons, the network size, or both, we conducted additional tests in which we varied the number of neurons and the network size independently (S7 Fig). We confirmed that the LRC ratio in the trained network did not change significantly as the number of neurons was varied (S7D Fig, n = 20, two-sided rank-sum test, n.s. p > 0.11). In contrast, the LRC ratio in the trained network increased as the network size was increased (S7 Fig, E, n = 20, two-sided rank-sum test, *p < 0.001). This result demonstrates that the emergence of LRCs mostly depends on the network size and is scarcely affected by the number of neurons.

Overall, these results suggest a possible mechanism to explain why LRCs exist species-specifically according to their cortex size—a large cortical network wired by sparse LRCs with dense local connection can be an optimal form to balance the performance and wiring cost, while LRCs cannot contribute to enhancing the performance enough to compensate for their wiring cost in a small network.

## Discussion

In the current study, we showed that cortical LRCs can play an important role in organizing a small-world network optimized for each size of the visual cortex and that this enables the cost-efficient recognition of visual stimuli. Our model simulations demonstrated that a combination of sparse LRCs and dense local connections compose a small-world network in a large network, with this enhancing the image classification performance accordingly. The network-size-dependent effect of LRCs could be predicted computationally, as the performance of the network is strongly correlated with the small-world coefficient of the network. This model analysis explains the species-specific existence of LRCs in animal data because the contribution of LRCs to enhancing the integration of visual information occurs only when the size of the network exceeds a certain threshold.

The ability to recognize natural scenes and objects is crucial for animals to survive. Natural images contain a significant portion of low-frequency components [31] and thus any visual circuit should be able to integrate this “global” information [40]. This requires sufficiently deep hierarchical structures with a large number of connections unless the convergent range of each feedforward projection is very large [41], comparable to that of a fully connected network. Previous observations in the brain have shown that this condition is not found experimentally. Moreover, due to the restricted volume of the physical space and limited metabolic resources in the brain, the visual pathway cannot develop such a deep structure comparable to conventional artificial intelligence models. These restrictions make it difficult to integrate global information of natural visual inputs only with feedforward convergent projections that are localized tightly. In theory, implementing a wide range of convergences between the layers also may not be an appropriate solution because doing so may lead to a significant loss of high-frequency “local” information due to the large size of the receptive fields. Our theoretical analysis in the current study shows that the combination of dense local connections and sparse LRCs in the layers of shallow feedforward networks may enable the brain to address this issue by capturing both local and global information at a minimal wiring cost.

In the current model simulation, we used a network with convergent feedforward projections instead of the convolutional neural network (CNN) typically used for deep learning model studies focusing on visual functions [42,43]. A convergent network model is more suitable for investigating mechanisms of biological brains because it consists of more plausible architectures, such as localized feedforward projections and a segregated receptive field for each neuron, following the observation that each neuron in the primary visual cortex (V1) has a receptive field that matches a local area in the visual space via a localized retino-cortical afferent projection [36]. On the other hand, CNN models commonly adopt a weight-sharing structure in which each neuron encodes different parts of the visual space using exactly the same filter. This type of design takes advantage of the reduced level of calculation overall but does not allow us to study the biological mechanisms underlying the target function utilized by the network. The current study targets a particular component of biological brains, long-range lateral connections in cortical circuits, for there is no equivalent in typical CNN models. Thus, we performed our model simulations using a convergent feedforward network model, which enabled us to control and modulate the key components of the model network, such as the statistics of the lateral connections.

Similar to the current study, there have been previous model studies of the role of lateral connections in visual information processing [44–46]. In these cases, lateral connections were implemented as intra-layer connections that allow communication between neurons in the same hidden layer, appearing to enhance the performance of feedforward networks. However, these models only implemented lateral connections of a limited spatial range, comparable to the short local connections in the current study. Distinct from these approaches, we have been focusing on the possible role of LRCs [35] that are distinguished from the local connections of a short lateral spread. In previous works, we demonstrated that sparse LRCs enable cost-efficient object recognition under the physical constraint of the hierarchical depth. Subsequently, in the current study we show that such an effect of LRCs is due to the optimization of the circuit structure as described by a small-world network theory and that this theoretical framework provides a better understanding of the species-specific organization of cortical circuitry.

The small-world network is an intriguing mathematical structure studied extensively in a number of model studies of complex systems. For example, in machine learning studies, it has been suggested that a small world is an efficient architecture with which to perform visual tasks [47,48] that demand fewer training parameters, taking less time to achieve the desired level of accuracy compared to densely connected networks. Interestingly, studies in the field of neuroscience have suggested that the anatomic structure and the functional connectivity of the brain can be described as a small-world network [49–52]—an analysis of connectome data suggests that a small-world configuration is commonly observed in whole brain networks across various species. Particularly for cost-efficient wiring in the brain, a small-world network is considered to minimize the total wiring cost necessary for communication by distant neurons while maintaining the local interaction between adjacent neurons. In the current study, we argue that the advantage of a small-world structure is also observed on a different scale; distinct from most previous studies that consider the network wirings among the brain regions, our new results show that a small-world network at the single-neuron wiring level should also be studied to elucidate how the brain organizes a cost-efficient functional circuit in local brain areas, such as the primary visual cortex.

In the current study, we did not explicitly implement a highly detailed anatomy of biological brains for simplicity of the model. In particular, recurrence is not implemented in the current model, which may play an important role during the integration of continuous temporal information. Previous models with lateral connections [44–46] accounted for the temporal delay of a continuous input stimulus, by which feedback may result in multiple interaction loops. Currently, our model only considers inputs from static images and thus can only have one recurrent loop. In our subsequent studies, multiple recurrent loops would be introduced with a continuous video stimulus, which may enable us to examine the contribution of LRCs to the continuous integration of global information. It must be also noted that there are a number of other relevant factors that affect our results—such as the cell density, variance of the convergent projection range, the nonlinear magnification factor of retinotopy, and recurrent and feedback circuits. Although all of these factors may affect information processing and thus their detailed effects may need to be examined in subsequent studies, our model suggests that modulation of the network structure and changes in the performance by these factors can be predicted and understood theoretically by considering a key characteristic of the system: how these factors modulate the small-world structure of the circuit.

A fundamental question about the emergence of LRCs during brain development remains. It has been reported that LRCs develop without visual experience or training because they are observed before eye-opening [53,54], implying that LRCs emerge spontaneously from early internal activities of the brain. Regarding this issue, previous studies have reported that feedforward afferents from the periphery may play important roles in the development of early cortical circuits [55,56]. In particular, LRCs in sensory cortices can be induced by feedforward projections from early retinal activities [57]. In an earlier computational study by our group, it was suggested that cortical LRCs originate from early peripheral activities before eye-opening [58]; long-range horizontal connections in V1 emerge from spatiotemporally structured retinal waves generated spontaneously. Subsequent studies of this scenario along with an analysis of developing V1 areas in young animals may provide further support for our model.

Overall, our results suggest that cortical long-range connections enable the organization of small-world networks for cost-efficient visual recognition under biological constraints. This finding offers a simple but powerful model that explains the role of cortical long-range connections and the underlying mechanism of a biological strategy for species-specific evolution.

## Materials and methods

### A neural network model for image classification

To implement a simplified model of the pathway from the visual space to V1, we used a multilayer perceptron model consisting of three layers—an input layer (32 × 32 units (pixels)), a hidden layer (13 × 13 ~ 32 × 32 units), and a readout layer (13 × 13 ~ 32 × 32 units)–to perform the classification task. The number of neurons in the readout layer is set to be identical to that in the hidden layer so that their receptive fields cover the entire input visual space. We selected the sizes of 32x32 and 17x17 to represent the large and small network. This size was chosen by considering that (i) the ratio of the area between networks needed to be greater than the V1 ratio we wanted to test (2.33 times, rats vs. mice) for species with and without LRCs, and (ii) the number of units in a small network must be sufficient to perform a visual task equivalent to that in a large network. When networks of various sizes were generated, the density of neurons in the networks was fixed at 1 (e.g., Large network: size = number of neurons = 32x32; Small network: size = number of neurons = 17x17). This is based on biological observations that the V1 cell density is fairly consistent across species [28]. The schematic architecture of the model is shown in Fig 2A and 2B.

To match the model parameters with the statistics observed in the biological data, we assumed a condition in which 15° visual images were shown to a head-fixed animal such as tree shrew [5,27,59]. Considering the observed data of the magnification factor and the V1 size of various mammals (mice and tree shrews), the scaling factor was set to 0.1 mm/unit so that 0.1 mm in the cortical space of the biological data matched a one-unit distance of connections in the hidden layers. For example, the biological definition of the LRC as a lateral connection longer than 1 mm in the literature [60] was converted to that for model LRCs with connections longer than ten units.

The feedforward connectivity (FF) between the input and hidden layers was set to a local convergent structure following work by Hubel and Wiesel [36,61]. The i^th^ neuron in the hidden layer has a circular receptive field; that is

$$if\;d_{ij}<c,\;w_{ij}^{b}=1$$

$$otherwise,\;w_{ij}^{b}=0,$$

where j is the index of the neuron in the input layer and *d*_*ij*_ is the Euclidean distance between neurons i and j when the input layer is projected to the hidden layer. Here, c denotes the size of the receptive field, which is equivalent to the maximum distance *d*_*ij*_, which was initially set to four units. The term $w_{ij}^{b}$ is a Boolean parameter that represents the presence of a connection between neuron i and j.

Connections between the hidden layer and the readout layer were set to ensure a local convergent projection following the observation that retinotopy is maintained up to higher visual areas in the brain. Among the neurons in the readout layer whose receptive field contains the fixation center of visual space, N (the number of labels in the task) readout neurons were randomly selected and their activations were used for the classification task. We used readout neurons only covering the center of the visual space for our analysis to prevent any possible edge effect from neurons located around the edge of the hidden layer.

The intra-layer lateral connections link two neurons in a hidden layer. The length distribution of the lateral connections followed an exponential function fitted from data pertaining to the V1 of the tree shrew [5],

$$Y={0.28e}^{-1.48d},$$

where *d* is the connection length (converted to units) and *Y* is the connection probability. Connections were randomly generated with a probability of 0.5. Once the total number of lateral connections is determined, the weight matrix *W*_*LAT*_ is generated using the given length distribution. Here, all connection weights were initialized using a Gaussian distribution with a mean of 0 and a standard deviation of 0.05. The overall network computation is as follows:

$$H=ReLU(W_{LAT}W_{CON}X+W_{CON}X+b_{CON})$$

$$Y=softmax(W_{RO}H+b_{RO})$$

In this equation, *X* is the input pixel, *H* denotes the hidden layer activation, *Y* is the output layer activation, *W*_*CON*_ and *W*_*RO*_ denote the FF connection matrix of each layer, *b*_*CON*_ and *b*_*RO*_ are correspondingly the biases of the input and hidden layer, and *W*_*LAT*_ is the lateral connection matrix. The number of feedback loops by lateral connections was limited to one to investigate the immediate effect of LRCs.

To undertake image classification, the model was trained using stochastic gradient descent as a simplified model of the learning rule. During the learning process, *W*_*CON*_, *W*_*RO*_ and *W*_*LAT*_ in each layer were trained. The batch size was set to 512 images and the weight decay factor was set to 0. The learning rate was set to a constant value of 0.1. Other hyperparameters such as the number of epochs were selected to provide reasonable accuracy during the image classification task [62] (Number of training epoch = 500). All simulations were performed using the MATLAB deep learning toolbox.

### Image datasets

The model was trained to perform the image classification task. To investigate the frequency-specific role of LRCs for image classification, we generated three datasets by modifying the size and position of hand-written digits (MNIST). Details are as follows:

1. The “shape” dataset was designed by resizing a hand-written digital image to 5 × 5 pixels in the center of 32 × 32 pixels. The “local” dataset consists of four categories depending on the number (four digits selected from 0 to 9). All combinations of digits were used for stimulus variation. For this dataset, only the local information (shape) of the digits was required for classification.
2. The “position” dataset was created using the following procedure. First, the 32 × 32 pixel area was divided into four 16 × 16 areas. Second, one Gaussian dot was generated in one of the quadrants. The size and the distance from the center were control parameters. This dataset also consisted of four categories depending on the location of the dot.
3. The “both” dataset was generated by simply overlapping the “digit” dataset and the “position” dataset. The “both” dataset consisted of sixteen categories depending on the types and locations of the digits.

For the “shape” dataset, the local profile of the digits was the only information used for classification. For the “position” dataset, the shape of the numbers is irrelevant and only the positions of the dots served as information for classification. For the “both” dataset, the network should perceive both the shape and position of the digits in order to classify the images correctly. For all three datasets, 10,000 images were generated for the training dataset and 2,000 newly generated images were used for the test dataset.

### Network rewiring process

To examine the exact role of a long-range lateral connection (≥ 10 units), we varied the ratio of the LRCs in the lateral connections and tested the image recognition performance after training of the modified MNIST dataset (shape, position, both). For a fair comparison of the performances of networks with different structures, the total number of connections was controlled because it is obvious that the network with more learnable parameters would perform better. To keep the number of learnable parameters the same while varying the network connectivity, we ablated the local lateral connections (< 10 units) in the hidden layer while adding the same number of LRCs. The feedforward connections were not ablated because those connections were thought to be critical for the classification.

### Network connectivity indices

Overall, the network parameters used in the paper follow earlier definitions [33,34] with small modifications. The clustering coefficient *C* is the average density of connections between the neighbors and is described as

$$C=log(\frac{1}{N_{hidden}}\sum_{j}c_{j}),$$

where *N*_*hidden*_ is the number of hidden neurons and *c*_*j*_ is the local coefficient of *j* hidden neurons. Here, *c*_*j*_ is determined by the following equation:

$$c_{j}=\frac{1}{N_{neighbor,j}}(\sum_{i}\frac{1}{d_{ii\prime }}+\sum_{j^{\prime }}\frac{1}{d_{j\prime j\prime \prime }}),$$

where *N*_*neighbor*,*j*_ is the number of neurons connected with *j* hidden neurons, *d*_*ii*′_ is the inter-neighbor distance between the source *i* input neuron and destination *i*’ input neuron, which are connected via the *j* hidden neuron. Similarly, *d*_*j*′*j*"_ is the inter-neighbor distance between the source *j*′ hidden neuron and destination *j*′ hidden neuron, which are connected with the *j* hidden neuron.

The characteristic path length *L* is defined as shown below.


$$L=\frac{1}{N_{input}(N_{input}-1)}\sum_{i\neq i\prime }l_{ii\prime }$$

Here, *N*_*input*_ is the number of input neurons and *l*_*ii*′_ is the shortest path between *i* and *i*′. The term *l*_*ii*′_ is defined as the shortest path from a random input neuron I to another input neuron *i*′. It should be noted that the shortest path must be included for at least one hidden neuron because the network is a layered structure.

The small-world coefficient (SW) was defined as the ratio of the clustering coefficient C and path length L. However, this index is influenced considerably by certain network parameters, such as the number of units. To avoid this problem, first we defined the "regular" and the "random" networks with the same number of connections as the corresponding networks. These networks are used in the normalization process for a fair comparison between SW between networks with different total numbers of connections. The normalized small-world coefficient [32] was defined as follows:

$$\Delta C=\frac{C_{regular}-C}{C_{regular}-C_{rand}},$$

$$\Delta L=\frac{L-L_{rand}}{L_{regular}-L_{rand}},$$

$$SW=1-\sqrt{\frac{{\Delta C}^{2}+{\Delta L}^{2}}{2}},$$

where *C*_*rand*_ and *L*_*rand*_ are the clustering coefficient and characteristic path length on a random network consisting of only randomly generated lateral connections. The terms *C*_*regular*_ and *L*_*regular*_ are correspondingly the clustering coefficient and characteristic path length on a random network consisting of only local lateral connections.

### Gradient-based connectivity optimization and pruning

We examined how random initial connectivity would evolve by training to minimize the total connection length while maximizing the image classification performance. We used the gradient-based optimization method that penalized the connection length used in our previous study [35]. The objective function consists of a classification error minimization term and a connection length-penalty term, as follows:

$${L=argmin}_{W}\left((1-B)\frac{1}{N}\sum_{i=1}^{N}L_{i}(f(x_{i},W_{lat}),y_{i})+B\sum_{k}\sum_{l}\frac{{Len}_{k,l}⨂W_{k,l}^{2}}{2}\right)$$

where *W*_*lat*_ is the weight matrix of the network, N is the number of connections, *Len*_*k*,*l*_ represents the connection length matrix, k represents each layer, and *B* represents the ratio between error minimization and the length penalty. The value of *B* was chosen so that small changes in both terms can modulate the loss function significantly. The stochastic-gradient descent method was applied for optimization. To simulate the spontaneous pruning of unnecessary connections during the training epochs, we disconnected connections with a weight value below 0.05 after the training step.

## Supporting information

S1 Fig Species-specific existence of long-range horizontal connectivity in V1. (A). Distribution of the connection lengths of lateral connections on a scale normalized to the size of each V1. Note that data from species with no LRCs (i.e., mouse) is differentiable from those of species with LRCs (i.e. Tree Shrew) even on this relative scale. (B). Visualization of each type of V1 connectivity in mice and tree shrews (adapted from Seeman 2018 and Bosking 1997). Dashed black circles indicate a connection boundary shorter than 1 mm, and dotted red circles indicate a connection boundary scaled to 10% of the V1 size.
(TIF)

S2 Fig Variations of parameters in the “position” and “shape” stimulus datasets. (A). The center-dot distance *d* of the “position” dataset was varied and the classification accuracy rates of the network with 0% and 30% LRCs were measured. Note that the performance of the network without LRCs (LRC 0%) decreased significantly as *d* was increased, whereas that of the network with LRCs (30%) was fairly consistent. (B). The classification performance for “position” stimulus increases and also becomes less vulnerable to variations of the stimulus condition as the LRC ratio increases. (C). The “shape” dataset consists of four numbers selected from 0 to 9. All possible combinations of digits (210 in total) were tested. (D). The network with LRCs (LRC 30%) showed lower performance than that of the network without LRCs (LRC 0%).
(TIF)

S3 Fig Variations of parameters in the “both” stimulus dataset. (A). The center-dot distance and the combination of digits selected were varied in the “both” stimulus dataset. (B). The small-world coefficient (SW) and classification accuracy of the networks were measured with variations of the stimulus parameters and the LRC ratio. (C) Normalized accuracy. The maximum accuracy was set to 1 for each curve. (D). In a fairly large parameter regime of the stimulus variation, a significant correlation was observed between the classification performance accuracy and the SW of the network.
(TIF)

S4 Fig Choice of balancing parameters between two terms of the loss function: the classification error and the length penalty. The total length of lateral connections and the classification accuracy were both estimated while the balancing factor *B* was varied (Top). The balancing factor *B* was selected to maximize the performance-cost ratio—maximizing the classification accuracy while minimizing the total length of the connections (Bottom).
(TIF)

S5 Fig Emergence of LRCs for various types of visual stimuli. (A). Networks were trained and pruned with the “Shape,” “Position,” and “Both” datasets. (B). For the datasets containing “global” information (“Position” and “Both”), a certain portion of LRCs survived after training. Note that the ratio of LRCs decreases sharply at the early stage of training but later converges asymptotically to a constant value. (C). Distribution of the lengths of lateral connections after training with each dataset. (D). Comparison of the LRC ratio after training between each case. Note that LRCs survived only when the input data contained “global” information (only for the “position” and “both” and not for the “shape” dataset).
(TIF)

S6 Fig Robust emergence of long-range connections for a balance between performance and the wiring cost across structural variations. (A). Variations of the network structural parameters for the network size and connection density. The network size was varied from 17 x 17 to 32 x 32. (B). Change of the LRC ratio during training in three network sample sizes (17x17, 25x25, and 32x32). (C). The connection density represents the ratio between the number of initial wirings and the maximum number of wirings possible. This density was varied from 0.25 to 1, where 1 represents the condition in which all possible pairs of units in the network were wired initially. (D). Change of the LRC ratio during training in three sample density values (Connection density = 0.75, 0.5, and 0.25). (E) Distribution of the observed lateral connection length after training. (F) Monotonic increasing trend of the LRC ratio with the network size (n = 20, two-sided rank-sum test, *p < 0.01). Error bars represent the standard deviation for 20 repeated trials.
(TIF)

S7 Fig Emergence of LRCs depending on the size of the network but not on the number of neurons. (A). Correlation between the number of neurons and the network size used for analysis in S6 Fig (B). The number of neurons and network size were varied independently in the new simulation. The number of neurons was modified while maintaining a constant network size (Case 1: number = 25x25, 23x23, and 19x19; size = 25x25). In this case, neurons were randomly chosen and removed from the initial network. Similarly, the network size was varied while keeping the number of neurons constant (Case 2: size = 25x25, 27x27, and 31x31; number = 25x25). (C). Change of the LRC ratio during the training of sample networks (size = 25x25; number = 25x25). (D). No significant change of the LRC ratio was observed when varying the number of neurons (n = 20, two-sided rank-sum test, n.s. p > 0.11). (E). The LRC ratio increased when the network size was varied (n = 20, two-sided rank-sum test, *p < 0.001). Error bars represent the standard deviation for 20 repeated trials.
(TIF)
