# Supplementary figures and images for: Species-specific wiring of cortical circuits for small-world networks in the primary visual cortex
Source: PLoS Comput Biol. 2023 Aug 4;19(8):e1011343. doi: 10.1371/journal.pcbi.1011343 (PMC10403141; doi:10.1371/journal.pcbi.1011343)

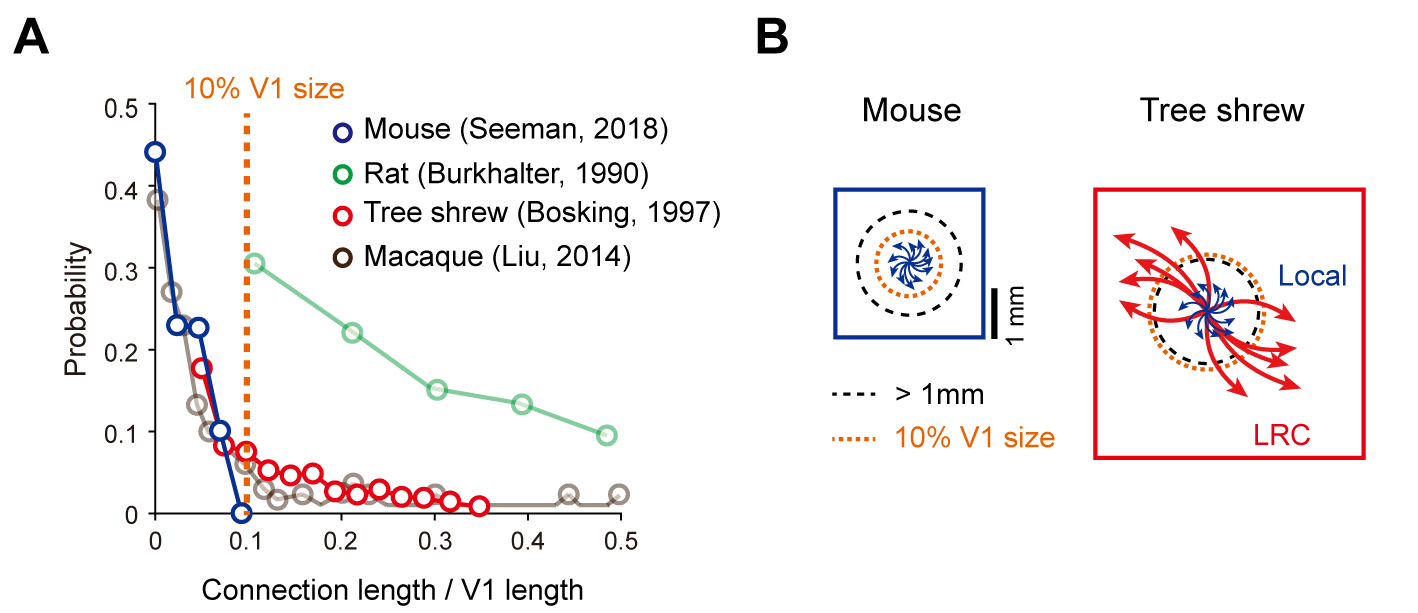

Supplement: S1 Fig — (A). Distribution of the connection lengths of lateral connections on a scale normalized to the size of each V1. Note that data from species with no LRCs (i.e., mouse) is differentiable from those of species with LRCs (i.e. Tree Shrew) even on this relative scale. (B). Visualization of each type of V1 connectivity in mice and tree shrews (adapted from Seeman 2018 and Bosking 1997). Dashed black circles indicate a connection boundary shorter than 1 mm, and dotted red circles indicate a connection boundary scaled to 10% of the V1 size. (TIF) [file pcbi.1011343.s001.tif]

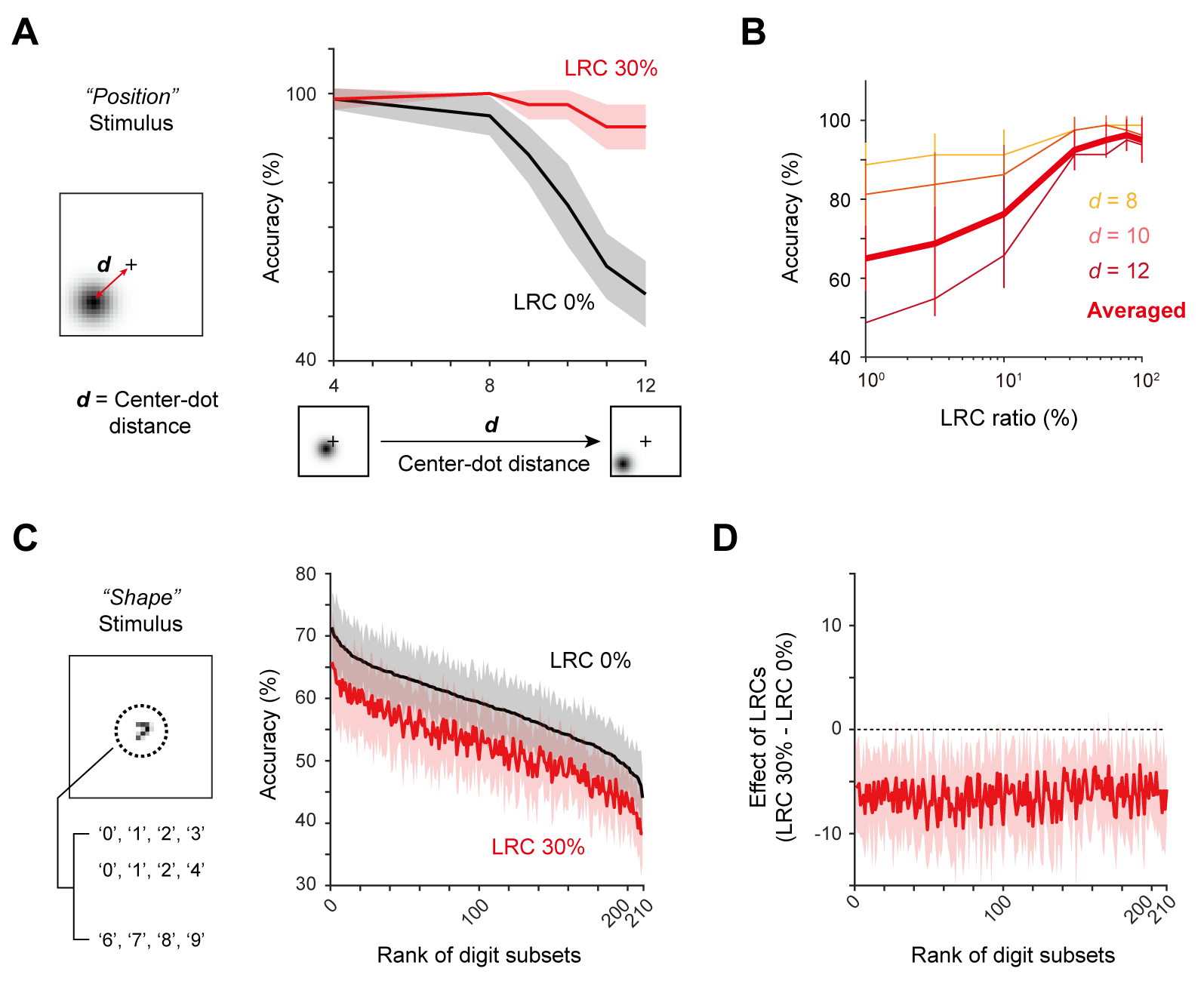

Supplement: S2 Fig — (A). The center-dot distance d of the “position” dataset was varied and the classification accuracy rates of the network with 0% and 30% LRCs were measured. Note that the performance of the network without LRCs (LRC 0%) decreased significantly as d was increased, whereas that of the network with LRCs (30%) was fairly consistent. (B). The classification performance for “position” stimulus increases and also becomes less vulnerable to variations of the stimulus condition as the LRC ratio increases. (C). The “shape” dataset consists of four numbers selected from 0 to 9. All possible combinations of digits (210 in total) were tested. (D). The network with LRCs (LRC 30%) showed lower performance than that of the network without LRCs (LRC 0%). (TIF) [file pcbi.1011343.s002.tif]

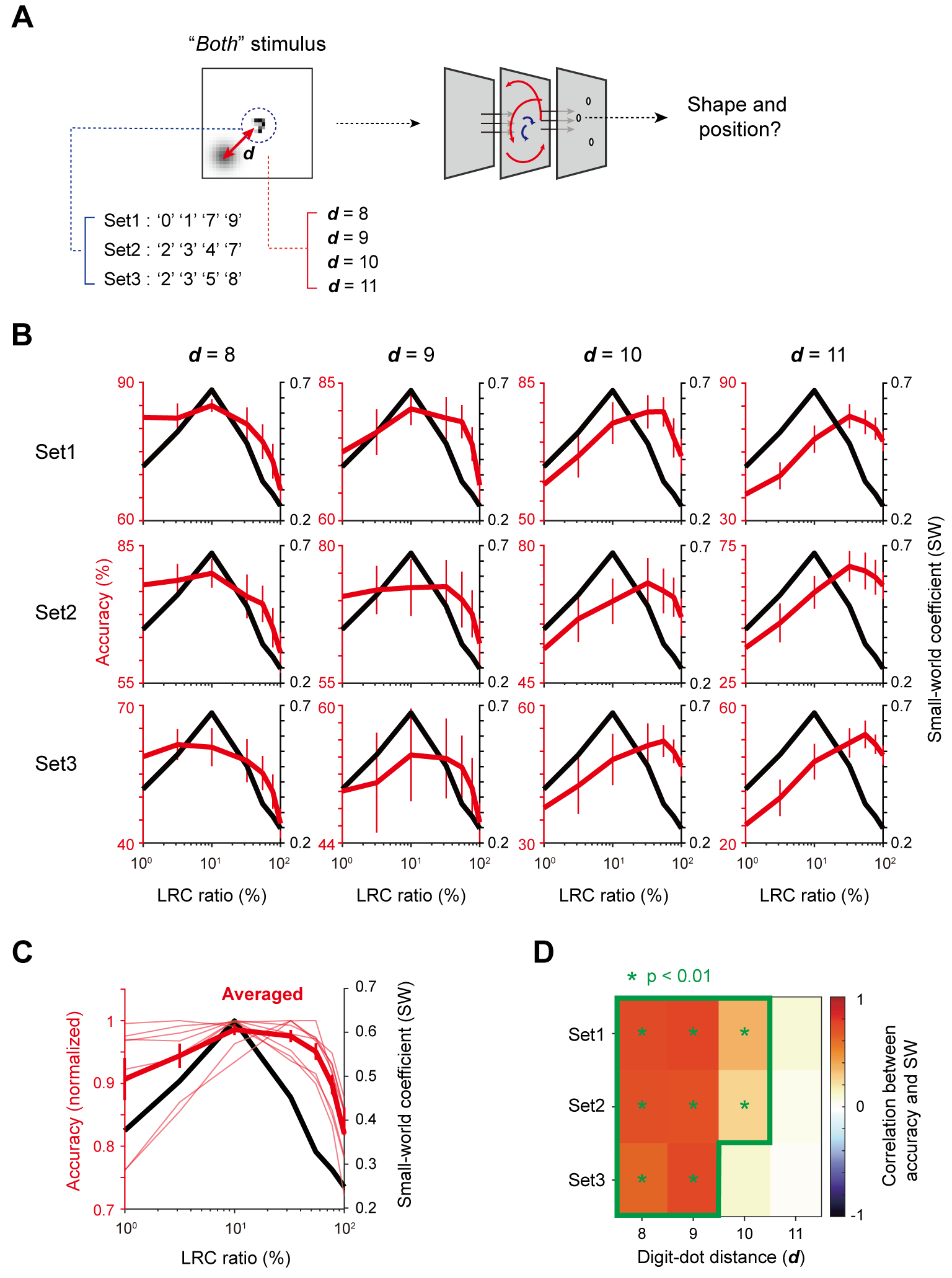

Supplement: S3 Fig — (A). The center-dot distance and the combination of digits selected were varied in the “both” stimulus dataset. (B). The small-world coefficient (SW) and classification accuracy of the networks were measured with variations of the stimulus parameters and the LRC ratio. (C) Normalized accuracy. The maximum accuracy was set to 1 for each curve. (D). In a fairly large parameter regime of the stimulus variation, a significant correlation was observed between the classification performance accuracy and the SW of the network. (TIF) [file pcbi.1011343.s003.tif]

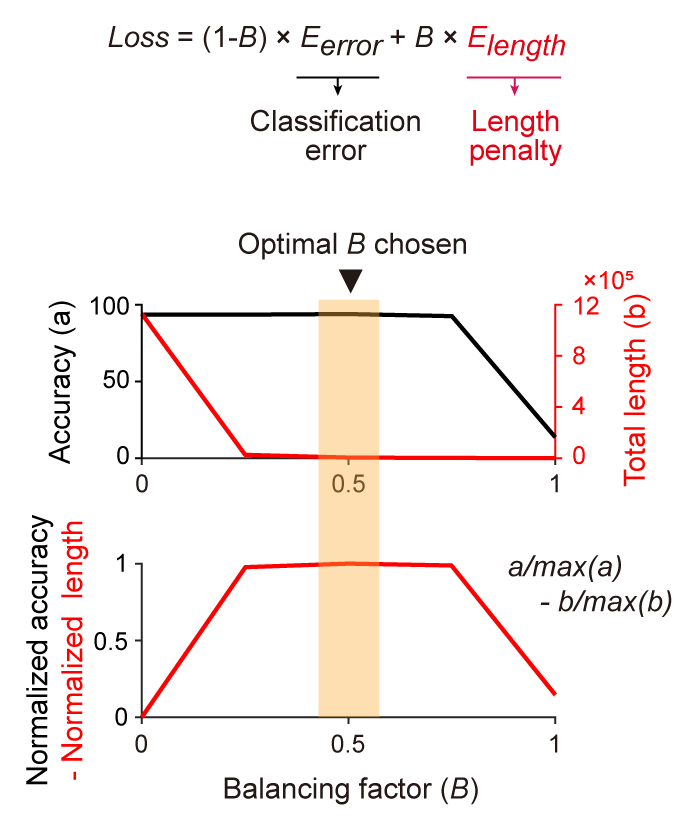

Supplement: S4 Fig — The total length of lateral connections and the classification accuracy were both estimated while the balancing factor B was varied (Top). The balancing factor B was selected to maximize the performance-cost ratio—maximizing the classification accuracy while minimizing the total length of the connections (Bottom). (TIF) [file pcbi.1011343.s004.tif]

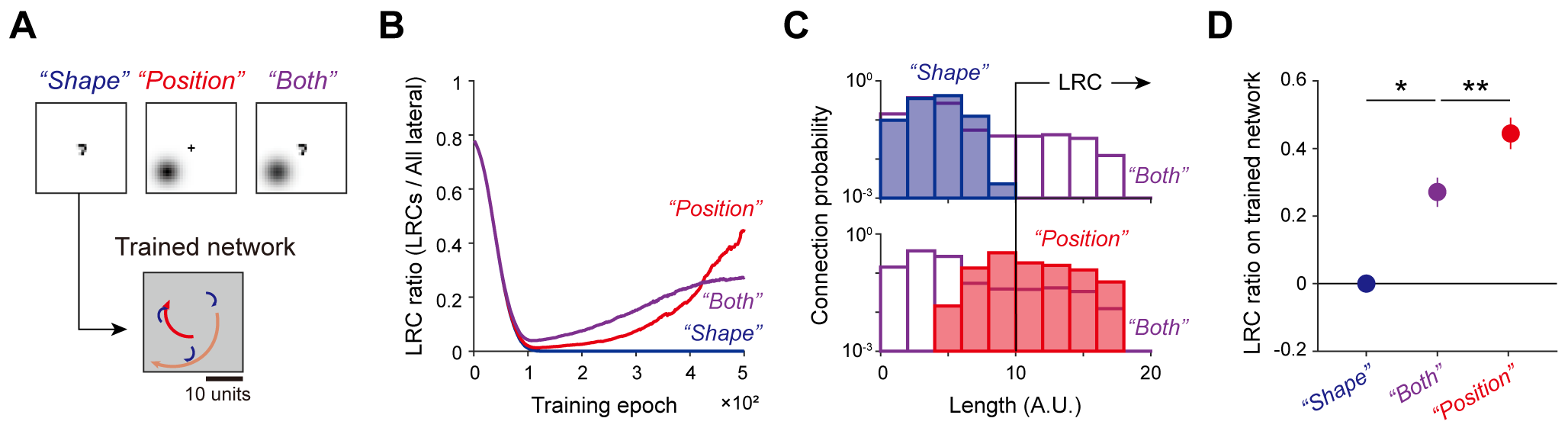

Supplement: S5 Fig — (A). Networks were trained and pruned with the “Shape,” “Position,” and “Both” datasets. (B). For the datasets containing “global” information (“Position” and “Both”), a certain portion of LRCs survived after training. Note that the ratio of LRCs decreases sharply at the early stage of training but later converges asymptotically to a constant value. (C). Distribution of the lengths of lateral connections after training with each dataset. (D). Comparison of the LRC ratio after training between each case. Note that LRCs survived only when the input data contained “global” information (only for the “position” and “both” and not for the “shape” dataset). (TIF) [file pcbi.1011343.s005.tif]

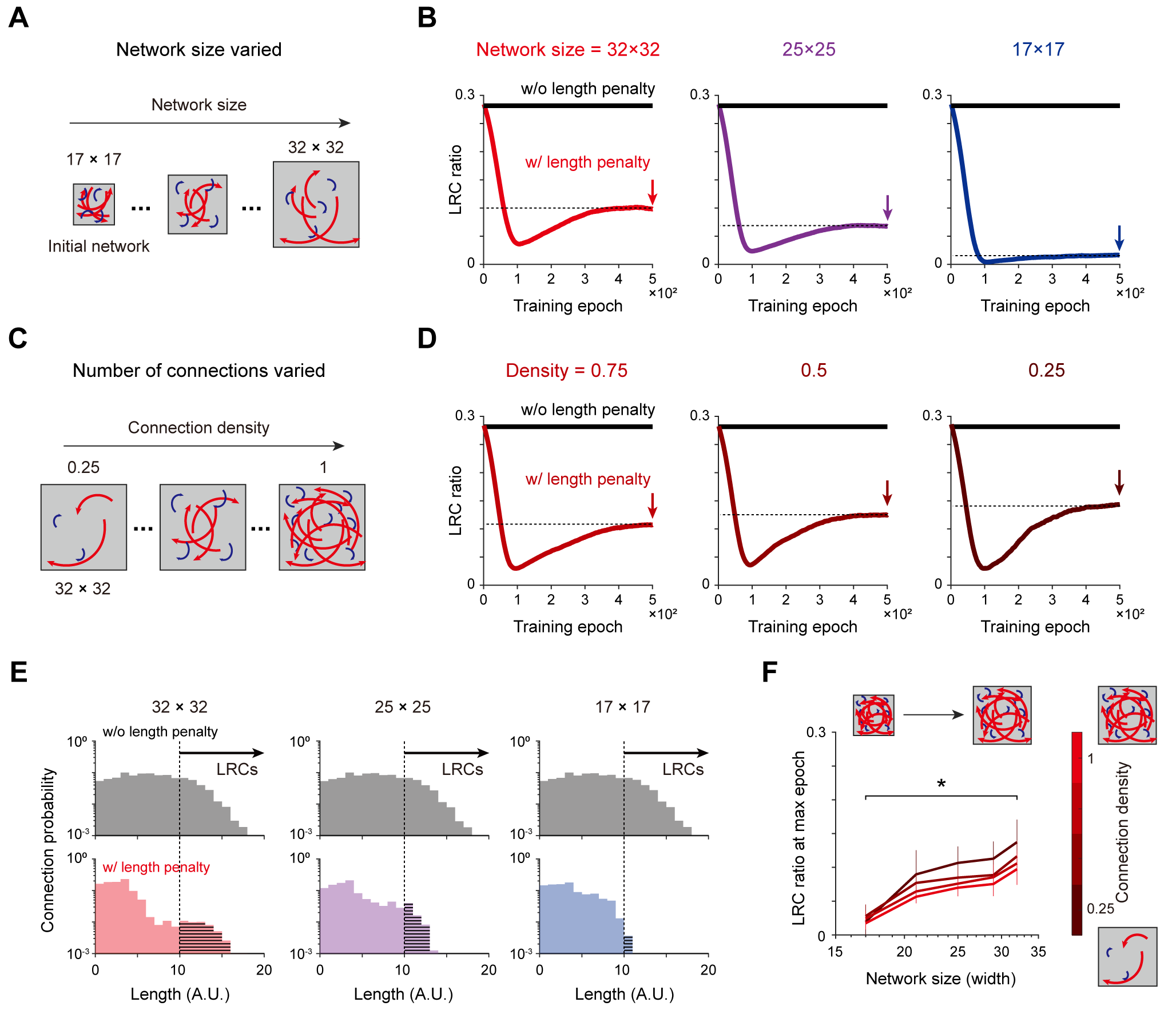

Supplement: S6 Fig — (A). Variations of the network structural parameters for the network size and connection density. The network size was varied from 17 x 17 to 32 x 32. (B). Change of the LRC ratio during training in three network sample sizes (17x17, 25x25, and 32x32). (C). The connection density represents the ratio between the number of initial wirings and the maximum number of wirings possible. This density was varied from 0.25 to 1, where 1 represents the condition in which all possible pairs of units in the network were wired initially. (D). Change of the LRC ratio during training in three sample density values (Connection density = 0.75, 0.5, and 0.25). (E) Distribution of the observed lateral connection length after training. (F) Monotonic increasing trend of the LRC ratio with the network size (n = 20, two-sided rank-sum test, *p < 0.01). Error bars represent the standard deviation for 20 repeated trials. (TIF) [file pcbi.1011343.s006.tif]

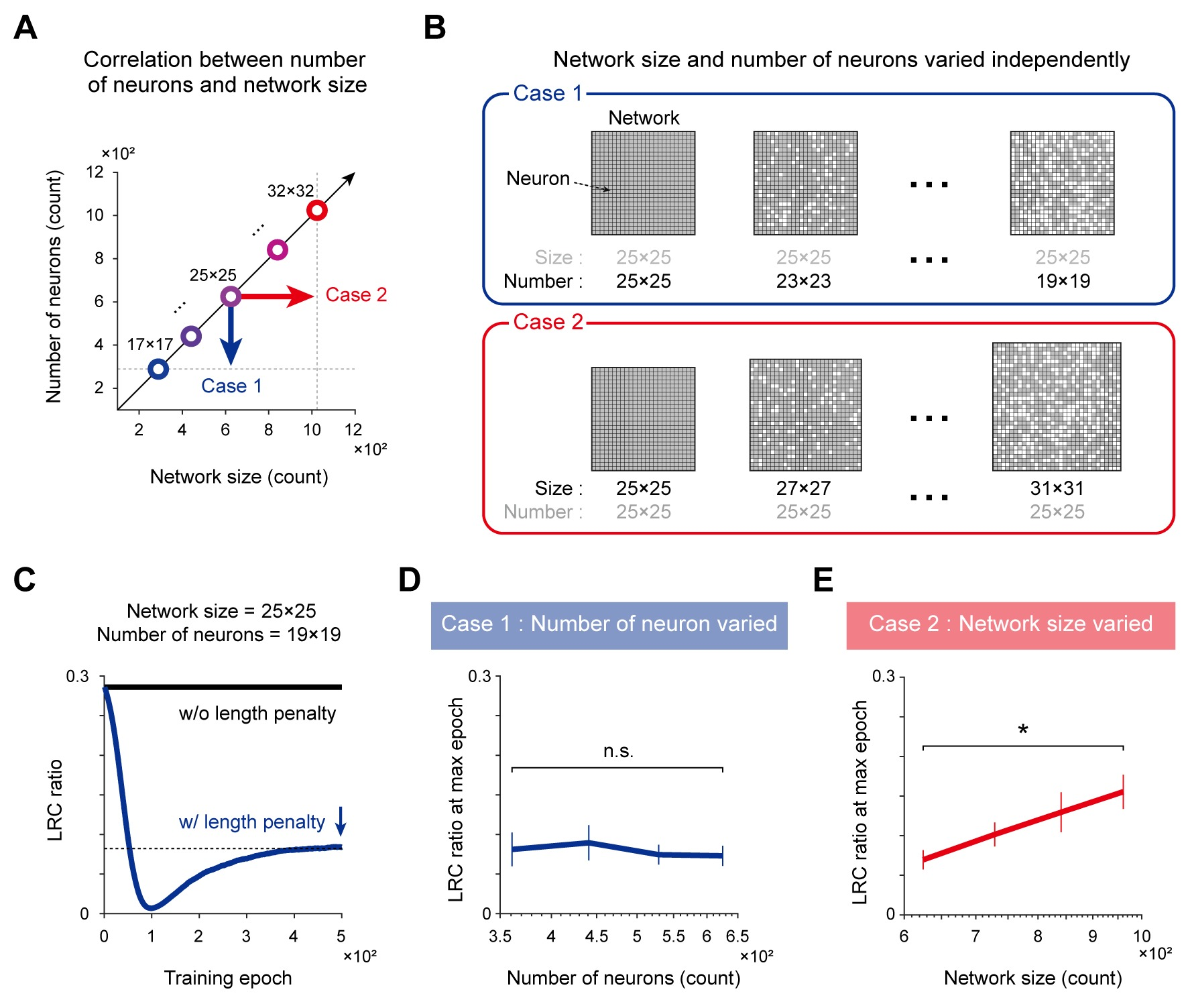

Supplement: S7 Fig — (A). Correlation between the number of neurons and the network size used for analysis in S6 Fig (B). The number of neurons and network size were varied independently in the new simulation. The number of neurons was modified while maintaining a constant network size (Case 1: number = 25x25, 23x23, and 19x19; size = 25x25). In this case, neurons were randomly chosen and removed from the initial network. Similarly, the network size was varied while keeping the number of neurons constant (Case 2: size = 25x25, 27x27, and 31x31; number = 25x25). (C). Change of the LRC ratio during the training of sample networks (size = 25x25; number = 25x25). (D). No significant change of the LRC ratio was observed when varying the number of neurons (n = 20, two-sided rank-sum test, n.s. p > 0.11). (E). The LRC ratio increased when the network size was varied (n = 20, two-sided rank-sum test, *p < 0.001). Error bars represent the standard deviation for 20 repeated trials. (TIF) [file pcbi.1011343.s007.tif]
